# Supplementary material for: Normative reference values of the handgrip strength for the Portuguese workers
Source: PLoS One. 2020 Aug 5;15(8):e0236555. doi: 10.1371/journal.pone.0236555 (PMC7406054; doi:10.1371/journal.pone.0236555)
Supplement: S1 Table — (DOCX) [file pone.0236555.s001.docx]

| Table 1 descriptive analysis of height for age groups in women and men. | | | | |
| --- | --- | --- | --- | --- |
|  | Min | Max | *x* | ±SD |
| Women |  |  |  |  |
| 20-24 | 153 | 174 | 161 | 5,47 |
| 25-29 | 147 | 177 | 162 | 6,81 |
| 30-34 | 152 | 179 | 162 | 5,50 |
| 35-39 | 150 | 176 | 161 | 6,06 |
| 40-55 | 148 | 170 | 158 | 5,80 |
| Men |  |  |  |  |
| 20-24 | 163 | 193 | 175 | 5,83 |
| 25-29 | 157 | 193 | 174 | 6,59 |
| 30-34 | 164 | 190 | 174 | 5,86 |
| 35-39 | 161 | 187 | 174 | 5,91 |
| 40-44 | 158 | 193 | 171 | 7,37 |
| 45-57 | 154 | 186 | 172 | 6,99 |
